# Performance Measures for Geometric Fitting in the NIST Algorithm Testing and Evaluation Program for Coordinate Measurement Systems

**DOI:** 10.6028/jres.100.042

**Published:** 1995

**Authors:** Theodore H. Hopp, Mark S. Levenson

**Affiliations:** National Institute of Standards and Technology, Gaithersburg, MD 20899-0001

**Keywords:** algorithm testing service, computational metrology, coordinate measurement systems, dimensional metrology, geometric fitting, measurement uncertainty, mechanical tolerancing, orthogonal distance regression, software uncertainty

## Abstract

The Algorithm Testing and Evaluation Program for Coordinate Measurement Systems (ATEP-CMS) is a Special Test Service offered under the NIST Calibration Program. ATEP-CMS evaluates the performance of geometric fitting software used in coordinate measurement systems. It is a Special Test because it is a new type of NIST service, experimental in nature and unsupported by historical data. This report documents and explains the rationale of the performance measures used in ATEP-CMS and analyzes the uncertainties of those measures.

## 1. Introduction

This report is concerned with the performance of software used in coordinate measurement systems (CMSs) to evaluated the geometric characteristics of manufactured parts. The particular performance characteristics of interest are those that impact the uncertainty of measurement results produced by CMSs.

Inspection planners need quantitative measures of performance to develop uncertainty budgets and to evaluate the quality of measurement results. In support of this need, NIST has recently established a Special Test Service, the Algorithm Testing and Evaluation Program for Coordinate Measurement Systems (ATEP-CMS), to measure the performance of geometric fitting software used in CMSs [1]. This report documents and explains the performance measures used in ATEP-CMS.

Geometric fitting is the process of computing the representation parameters of a geometric element that in some sense best represents a set of point coordinate data. This representative geometric element is called the *substitute geometry* for the data points. A manufactured hole, for instance, is usually not perfectly cylindrical because the process that produced it can never be totally perfect. Inspection of the hole might involve measuring the coordinates of selected points on the surface of the hole, fitting a cylinder to the measured points to minimize the sum of squares of the orthogonal distances from the points to the cylinder, and comparing the position, orientation, and size of the fitted cylinder to the dimensions and tolerances of the part specification.

Many factors affect the accuracy of inspection procedures.^1^ This report focuses on one of these factors: how close the computed fit is to the intended, mathematically defined, substitute geometry. The performance measures used in ATEP-CMS quantify how well the software computes substitute geometries over a range of inspection problems.

The next section provides background information on the need for algorithm testing, how ATEP-CMS works, and criteria for performance measures. Section 3 presents details of the test methods used in ATEP-CMS for various types of geometric elements. Section 4 discusses a statistical interpretation of algorithm performance and explains the method used in ATEP-CMS to summarize and interpret the test results. Section 5 presents an uncertainty analysis of ATEP-CMS results.

## 2. Background

The drive to develop methods for testing CMS algorithms began in Germany in the early 1980s, when it became apparent that the software supplied with commercial coordinate measurement machines (CMMs) varied widely in quality [3, 4]. In the United States, the American Society of Mechanical Engineers established a task force on CMM software that is now designated B89.4.10. Work intensified in 1988 following widespread notification within the defense industries of serious problems with certain commercial CMM software [5]. A U.S. standard on performance evaluation of CMS software is now in draft form and is expected to be issued for public review in early 1996 [6]. NIST’s ATEP-CMS service [1] supports this emerging standard.

### 2.1 What is Evaluated by ATEP

When discussing software, the term “testing” is used in many different ways, and it is important to understand the sense in which it is used within ATEP-CMS. It is specifically *not* used in the sense of software engineering, where software testing is aimed at supporting debugging or maintenance and typically involves code structure analysis, walk-throughs, and similar activities. Within ATEP-CMS, testing is strictly limited to black-box testing of how the actual behavior of the software compares with its intended behavior. Moreover, ATEP-CMS restricts itself to measuring the accuracy of reported results. Other performance characteristics—such as memory requirements, computing time, ease of use, and other factors—are not considered. In sum, ATEP-CMS ignores the fact that the software under test is software; the ATEP-CMS process would be unchanged if the fitting methods were implemented with an analog computer, as a digital filter circuit, or even mechanically [7].

ATEP-CMS does not address whether the intended behavior of the software is *appropriate* for a particular measurement task. That issue is not one of performance, but of whether a particular data analysis method is the right tool for the job. Such considerations are beyond the scope of a performance testing program.

ATEP-CMS currently supports testing of orthogonal distance regression fitting^2^ for seven geometry types: line, circle, plane, sphere, cylinder, cone, and torus. (Lines and circles are fit to three-dimensional point coordinates which need not be coplanar.) The reader is referred to Ref. [9] for information on how to use ATEM-CMS and the procedures used by NIST for conducting a test. Section 3 below describes the specific difference parameters used in ATEP-CMS for each geometry type.

### 2.2 Criteria for Performance Measures

In developing performance measures, the question naturally arises as to how one might choose one measure over another. One approach is to seek a reliable method of certifying software as “good” or “bad.” (This is the approach taken, for instance, by the German testing program [3].)

The approach taken in ATEP-CMS is somewhat different. We start with the observation that software is not mathematically perfect. (If nothing else, the end results must be represented in finite precision.) So rather than developing a pass/fail criterion, ATEP-CMS quantifies the expected performance numerically. The goal is to develop numerical measures of performance that provide to a user of the software, information necessary to decide whether the software is adequate for a particular application. To this end, we have used the following criteria in developing ATEP-CMS performance measures:

- each measure should be directly related to inspection tasks;
- each measure should combine like a standard deviation with other sources of uncertainty in a CMS;
- each measure should have reasonable probability and coverage interpretations;
- an estimate of uncertainty should be derivable for each measure.

The first three criteria directly support the goal of using the performance measures to establish uncertainty budgets for CMSs. The last criterion reflects the view that an assessment of software uncertainty is itself a measurement. By NIST [10] and international [11] guidelines, a quantitative statement of uncertainty should be part of any complete measurement result. The last criterion says that the ATEP-CMS performance measures must be amenable to such a treatment.

## 3. ATEP-CMS Test Methods

To start a software test, NIST generates a collection of data sets for each geometry type, consisting of three-dimensional point coordinates together with some book-keeping information. NIST also generates a fit for each data set, called the *reference fit* for that data set. Within ATEP-CMS, the reference fits are generated by fitting the data sets using NIST-developed fitting algorithms.^3^ (It is also possible to start with the desired fits and generate data sets having those fits as answers [13].)

The data sets are then sent to the software under test, which generates a *test fit* for each data set. The test fits, represented by a set of parameter values in a standard format, are returned to NIST for evaluation with respect to the corresponding reference fits.

This section describes how differences between a test fit and the corresponding reference fit are evaluated in ATEP-CMS. Differences between each pair of fits are represented by a set of *difference parameters.* Each geometry type gives rise to its own set of difference parameters. The relationship between the difference parameters and tolerance applications is also discussed.

### 3.1 General Procedure for a Single Fit

Methods for comparing one fit to a reference are described in the draft B89.4.10 standard [6]. The procedure for all geometry types follows a common pattern.

First, the test fit is bounded by projecting the data points onto the geometry. (The way this is done depends on the geometry type, and will be described below. Also, bounding does not apply to circles, spheres, or tori, which are naturally bounded.) This is done because tolerancing standards (e.g., Ref. [14]) specify that tolerances are to be evaluated over the full extent of the associated geometric features. ATEP-CMS assumes that the data points represent the features.

Once the test fit is bounded, geometric differences between the test fit and the reference fit are determined. Again, the specifics depend on the geometry type. In general, however, the differences are designed either to directly reflect a tolerancing application or to provide diagnostic information about the software under test. The difference parameters depend only on the geometry represented by the fit parameters, and not on the parameterization of that geometry.

Finally we note that in many cases the difference parameters are not symmetric. This is a result of the asymmetric treatment of the test and reference fit (the test fit is bounded; the reference fit is not). Thus, reversing the roles of test and reference fit will generally change the difference parameter values.

### 3.2 Application to Specific Geometry Types

The remainder of this section discusses the application of the general procedure to the seven geometry types currently supported by ATEP-CMS: lines, circles, planes, spheres, cylinders, cones, and tori. For each geometry type, we describe the fitting objective, typical uses for such fits in inspection, and the difference parameters computed within ATEP-CMS.

#### 3.2.1 Lines

Line fitting minimizes the sum of squares of orthogonal distances from the points to the fit line. Line fitting is commonly used to check straightness and to establish an axis from the centers of circular cross sections. In the latter case, the axis may be checked for location or orientation or used as a coordinate datum axis.

Within ATEP-CMS, two parameters representing differences between the lines are computed. (Henceforth, we will call such parameters, *difference parameters.*) One difference parameter is the angle between the test and reference lines. This directly relates to the use of the fit as a datum axis. To define the other parameter, the test fit line is first trimmed to a segment by the orthogonal projection of the data points. Then the second parameter is the maximum orthogonal distance from the segment endpoints to the reference line. This is a measure of separation between the lines and directly relates to the assessment of straightness, position, and orientation. Both parameters are inherently nonnegative quantities. (Henceforth, quantities that are inherently nonnegative will be referred to as *magnitudes.*)

#### 3.2.2 Circles

Circle fitting is a two-step process. First, the plane of a circle is found by fitting a plane to the data (as described below). Then, the points are projected into the plane and an orthogonal distance regression circle is fit to the projected points. Note that this process generally results in a different circle than would be found by using the three-dimensional distances from the points to the circle.

Circle fitting is commonly used to check roundness, runout, cross-sectional size, the straightness of the median line of a hole or shaft, and sometimes position. It is also sometimes used as one step in finding an axis. The purpose of the two-step fitting process is that, in practice, the deviations of the points from a plane usually result from measurement error only, while the deviations from a circle in the plane also include the effects of form deviation of the part.

Within ATEP-CMS, three difference parameters are computed. One is the angle between the planes of the test and reference circles. This is primarily a diagnostic measure^4^ that can explain other differences between the fits. The second parameter is the Euclidean distance between the circle centers, measured in three dimensions. This relates directly to assessment of roundness, position, and establishing an axis. The third parameter is the difference between the test and reference circle radii. This relates directly to the assessment of size. The first two parameters are magnitudes, while the last parameter is a signed quantity, and is negative whenever the test fit radius is smaller than the reference fit radius.

#### 3.2.3 Planes

Plane fitting minimizes the sum of squares of orthogonal distances from the points to the fit plane. Plane fitting is commonly used to establish a coordinate datum plane and to check flatness, position, and orientation.

Within ATEP-CMS, two difference parameters are computed. One parameter is the angle between the test and reference planes. This directly relates to the use of the fit as a datum plane. To define the second parameter, the data points are orthogonally projected onto the test fit plane. The convex hull of the projected points in the plane forms a planar patch. The second parameter is the maximum separation of the planar patch measured orthogonally from the reference plane. This measure of separation directly relates to the assessment of flatness, position, and orientation. Both parameters are magnitudes.

#### 3.2.4 Spheres

Sphere fitting minimizes the sum of squares of orthogonal distances from the points to the fit sphere. Sphere fitting is commonly used to check form, size, and location, and occasionally to locate the origin of a coordinate system.

Within ATEP-CMS, two difference parameters are computed. The first parameter is the Euclidean distance between the sphere centers. This relates directly to assessment of form, position, and establishing an origin. The second parameter is the difference between the test and reference sphere radii. This relates directly to the assessment of size. The first parameter is a magnitude, while the second is a signed quantity, and is negative whenever the test fit radius is smaller than the reference fit radius.

#### 3.2.5 Cylinders

Cylinder fitting minimizes the sum of squares of orthogonal distances from the points to the fit cylinder. Cylinder fitting is commonly used to check cylindricity and size, and to establish an axis. In the latter case, the axis is used to check position and orientation and to establish a coordinate datum.

Within ATEP-CMS, three difference parameters are computed. One parameter is the angle between the test and reference cylinder axes. This directly relates to the use of the fit as a datum axis. To define the second parameter, the test fit axis is first trimmed to a segment by the perpendicular projection of the data points. Then the second parameter is the maximum perpendicular distance from the segment endpoints to the reference cylinder axis. This is a measure of separation between the axes and directly relates to the assessment of cylin-dricity, position, and orientation. The third parameter is the difference between the test and reference cylinder radii. This relates directly to the assessment of size. The first two parameters are magnitudes, while the third is a signed quantity, and is negative whenever the test fit radius is smaller than the reference fit radius.

#### 3.2.6 Cones

Cone fitting minimizes the sum of squares of orthogonal distances from the points to the cone surface. Cone fitting is commonly used to check profile and conical taper.

Within ATEP-CMS, four difference parameters are computed. One is the angle between the test and reference cone axes. This directly relates to the use of the fit for checking profile. To define the next parameter, the test fit axis is first trimmed to a segment by first projecting the data points perpendicularly onto the cone surface and then orthogonally projecting the projections onto the cone axis. Then the second parameter is the maximum perpendicular distance from the segment endpoints to the reference cone axis. This is a measure of separation between the axes and relates to the assessment of profile. To define the third parameter, the reference cone axis is bounded using the same process as was used for the test fit. For each fit, the perpendicular distance from the midpoint of the axis segment to the corresponding cone surface is a measure of location of the cone along its axis. The third parameter is the difference between the location of the test fit cone and the location of the reference fit cone along their respective axes. This is primarily diagnostic, and relates to the assessment of profile. The last (fourth) parameter is the difference between the test and reference cone half-angles. This relates directly to the assessment of conical taper. The first two parameters are magnitudes, while the last two are signed quantities, and are negative whenever the test fit cone parameter is smaller than that of the reference fit.

#### 3.2.7 Tori

Torus fitting minimizes the sum of squares of perpendicular distances from the points to the torus surface. Torus fitting is commonly used to check major and minor radii and profile. Occasionally torus fitting is used to establish a datum axis or plane.

Within ATEP-CMS, four difference parameters are computed. One is the angle between the test and reference torus planes. This directly relates to the use of the fit for checking profile and as a datum plane or axis. The second is the Euclidean distance between the torus centers. This also relates to the assessment of profile. The third and fourth parameters are the difference between the test and reference fit major and minor radii. These relate directly to the assessment of major and minor radius tolerances. The first two parameters are magnitudes, while the last two are signed quantities, and are negative whenever the test fit torus parameter is smaller than that of the reference fit.

## 4. Data Interpretation

The previous section described how each data set generates a set of difference parameters representing the differences between the test fit to the data set and the corresponding reference fit. In this section we discuss how these difference parameters are summarized into performance measures. For each difference parameter, we will define an associated performance measure summarizing the difference parameter values for all the data sets.

We consider the set of difference parameter values associated with each data set to be one (random) sample of software performance from a theoretical underlying population of inspection problems. We will use a statistical approach to the interpretation of the observed sample values. In Sec. 4.1 we derive the population characteristic we wish to use as a performance measure. In Sec. 4.2 we derive a practical estimator for that measure.

### 4.1 Statistical Model of Algorithm Behavior

We deal with each geometry type separately, and consider each performance measure (and associated difference parameters) independently. For simplicity, consider a single performance parameter for a given geometry type, as described in Sec. 3. Each data set represents an inspection problem drawn at random from a theoretical underlying population of inspection problems. We call the substitute geometry defined by the mathematical objective function the *true fit* to the data set. We assume the true fit cannot be computed exactly. For the *i*th data set, we now define three difference parameter values:

*t_i_*—the difference between the test fit and the true fit to the data set; *t_i_* is unknown and unobservable.
*r_i_*—the difference between the reference fit and the true fit to the data set; *r_i_* is unobservable, but it can be bounded using numerical analysis theory (see Sec. 5).
*p_i_*—the difference between the test fit and the reference fit to the data set; *p_i_* is the calculated difference parameter for the *i*th data set.

These quantities are random variables since they are functions of the data set, which is a random sample. As random variables, we can define various expectations. For the test fit quantities *t_i_* we define the mean

$$\mu_{T}=E\left(t_{i}\right);$$

the standard deviation

$$\sigma_{T}=\sqrt{E\left({\left(t_{i}-\mu_{T}\right)}^{2}\right)};$$

and the root-mean square error (rmse)

$$\gamma_{T}=\sqrt{E\left(t_{i}^{2}\right)},$$

where *E*() denotes the average over all the data sets—that is, the expected value taken over the theoretical population of inspection problems. We define analogous quantities for *r_i_*: *µ*_R_, *σ*_R_, and *γ*_R_; and for *p_i_*: *µ*_P_, *σ*_P_, and *γ*_P_.

If the *t_i_* are normally distributed, then one can make statements of coverage like

$$\mathrm{Pr}\left\{|t_{i}-\mu_{T}|\leq 2\sigma_{T}\right\}\approx 0.95.$$ (1)

This approach has two shortcomings. First, two parameters, *µ*_T_ and *σ*_T_, are needed to summarize the test; for simplicity, we would like a single measure of performance. Second, it is difficult to estimate *µ*_T_ and *σ*_T_, because we do not know the true fit.

If *µ*_T_ is less than half of *σ*_T_, then the rmse provides similar coverage.^5^

$$\mathrm{Pr}\left\{\left|t_{i}\right|\leq 2\gamma_{T}:\mu_{T}<\sigma_{T}/2\right\}\approx 0.95.$$

If *µ*_T_ > *σ*_T_/2, the actual coverage is greater (the software performance is better) than that suggested by a measure based on *γ*_T_.

The above coverage interpretations are valid for normal distributions. We believe that for the typical distributions that will arise, the coverage error will be on the conservative side since the tails are likely to be smaller than those of a normal distribution.^6^

ATEP-CMS uses *γ*_T_ as the theoretical performance measure of the software under test. Using the rmse overcomes one shortcoming of Eq. (1)—we have reduced the performance indicator to a single number. We have also replaced the problem of estimating *µ*_T_ and *σ*_T_ with the problem of estimating *γ*_T_. We consider this next.

### 4.2 Definition of Practical Performance Measures

In this section, we will describe two methods of estimating *γ*_T_. The first follows directly from the definition of *γ*_T_. The second is a simplified estimate. ATEP-CMS uses the second method, for reasons discussed at the end of this section.

We observe from the definitions that *t_i_* = *p_i_* + *r_i_* for signed parameters, and *t_i_* ≤ *p_i_* + *r_i_* for magnitudes. Then a simple substitution yields

$$\begin{array}{c} {\gamma_{T}}^{2}=E\left({t_{i}}^{2}\right)\\ \leq E\left({p_{i}}^{2}\right)+E\left({r_{i}}^{2}\right)+2E\left(p_{i}r_{i}\right)\\ \leq {\gamma_{P}}^{2}+{\gamma_{R}}^{2}+2\gamma_{P}\gamma_{R}. \end{array}$$

Thus, we can estimate an upper bound for *γ*_T_ if we can estimate *γ*_R_ and *γ*_P_. The upper bound can then be used as an estimate for *γ*_T_.

To estimate *γ*_R_, we need an estimate of the difference of each reference fit from the true fit. Methods for doing this will be discussed in detail in Sec. 5. For now, assume that the value of *r_i_* can be estimated as *u_i_*. Then an estimate^7^ ${\hat{\gamma }}_{R}$ of *γ*_R_ is the positive square root of

$${{\hat{\gamma }}_{R}}^{2}=\frac{1}{n}\sum_{i=1}^{n}u_{i}^{2},$$

where the sum is over all *n* data sets. (In this paper, all summations are from *i* = 1 to *n* unless otherwise indicated. Henceforth, we will use the summation sign alone with the index variable and limits understood.) Similarly, we estimate *γ*_P_ by the positive square root of

$${{\hat{\gamma }}_{P}}^{2}=\frac{1}{n}\sum {p_{i}}^{2}.$$

This first estimation method includes a term in the performance measure that represents the estimated difference between the reference and true fits.

A second method of estimating *γ*_T_ is simply ${\hat{\gamma }}_{T}={\hat{\gamma }}_{P}$, estimating *γ*_P_ as before. This method uses the reference fit as an estimate of the true fit. Unlike the first approach, the differences between the reference and true fits are not incorporated in the performance measure of the software under test. However, they will contribute to the uncertainty of the performance measures (discussed in Sec. 5).

An argument in support of the first method of estimation is that the second, simpler method may underestimate the true quantity. If the performance measures are to be used to establish uncertainty budgets for a CMS, use of the second method makes it critical to analyze the sensitivity of the budget to the uncertainties of the ATEP-CMS results. The first method may have smaller uncertainties.

On the other hand, there are three reasons why the simpler estimate is preferable. First, the software under test should not be penalized simply because the reference software has uncertain performance. Second, the first method may overemphasize the role of the reference software, and may be unduly pessimistic (since it is an upper bound). Finally, a sensitivity study is a proper precaution, and should be recommended anyway.

For these reasons, ATEP-CMS uses the second method—the observed sample rmse—to estimate the performance of the software under test. That is, for each performance measure in ATEP-CMS,

$${\hat{\gamma }}_{T}={\hat{\gamma }}_{P}=\sqrt{\frac{1}{n}\sum {p_{i}}^{2}}.$$

## 5. Uncertainty Analysis

This section addresses the uncertainty of the ATEP-CMS estimates of software performance. Recall that we have defined different performance measures for each geometry type, and that each performance measure is estimated from the corresponding set of difference parameters for the fits. We will show in this section that the uncertainty of each estimate arises mainly from two sources: (1) the uncertainty of the individual difference parameters due to the uncertainty of the reference fit and (2) the uncertainty arising from having sampled the population of inspection problems.

In Sec. 5.1 we will first examine the uncertainties of the reference fits themselves. In Sec. 5.2 we then examine how these uncertainties propagate to the difference parameters. Finally, in Sec. 5.3 we discuss how the difference parameter uncertainties combine with the sampling uncertainty to yield an overall uncertainty for each performance measure.

### 5.1 Uncertainty of Reference Fits

The uncertainties of the difference parameters for a single test fit arise primarily from the uncertainty due to the presence of (unknown) numerical errors in the reference fit.^8^ That is, the reference fit may not correspond exactly to the true fit. A detailed discussion of the NIST fitting algorithms and their uncertainties will be presented in a subsequent report. Here, we provide a summary of the pertinent results.

Our approach is to develop bounds for the numerical errors, assume the errors are uniformly distributed between the bounds, and combine the standard deviations of the errors into the uncertainty of the reference fits. The results presented in this section and the next are specific to the particular algorithms currently used in ATEP-CMS. It is possible that the algorithms may change in the future [16], in which case these results would no longer hold. (The performance measures would not change, however.)

#### 5.1.1 Centroid Coordinates

For all fits, the data are first translated to be centered at the origin. One source of uncertainty is the possible rounding error in computing the centroid. The ATS algorithms use the Kahan summation algorithm [17] with a C-language long double accumulator (80 bit IEEE extended real format). The error in the coordinates of the computed centroid can be bounded in terms of the unit roundoff *ε*_80_ for the accumulator (about 1.084×10^−19^). For instance, if *x*_c_ is the computed *x* coordinate of the centroid (the average of the coordinates *x_i_*, *i* = 1, …, *m*) and *x*_0_ is the true coordinate, then the following result holds:

$$\begin{array}{c} x_{c}=\frac{1}{m}\sum_{i=1}^{m}x_{i}(1+\delta_{i})+O(10^{-38})\sum_{i=1}^{m}\left|x_{i}\right|\\ \approx x_{0}+\frac{1}{m}\sum_{i=1}^{m}x_{i}\;\delta_{i}, \end{array}$$

where |*δ_i_*| ≤ 2*ε*_80_ ≈ 2.17×10^−19^ and in the second line we have ignored the term in *O*(10^−38^). Similar results hold for the *y* and *z* coordinates.

To conform to NIST policy on uncertainty statements, this must be converted to an uncertainty by assuming some distribution on the possible values of *δ_i_*. We will assume that the *δ_i_* are independent and uniformly distributed within the bounds [18]. Then the *i*th error term *x_i_δ_i_* is a random variable with mean of zero and standard deviation $2|x_{i}|\varepsilon_{80}/\sqrt{3}$. So, by the Central Limit Theorem [19], the *x* coordinate of the computed centroid of *m* data points is approximately normally distributed with mean *x*_0_ and standard deviation of

$$\frac{1.25\;\times \;10^{-19}\sqrt{\Sigma {x_{i}}^{2}}}{m}$$

Similar results hold for the *y* and *z* coordinates. For simplicity, the uncertainty of the centroid is modeled in ATEP-CMS by an isotropic, trivariate normal distribution using a standard deviation of

$$u_{0}=1.25\;\times \;10^{-19}\sigma_{\max}/m,$$

where

$$\sigma_{\max}=\max\left\{\sqrt{\Sigma {x_{i}}^{2}},\sqrt{\Sigma {y_{i}}^{2}},\sqrt{\Sigma {z_{i}}^{2}}\right\}.$$

#### 5.1.2 Linear Fits (Lines and Planes)

For lines and planes, orthogonal distance regression can be formulated as a linear algebra problem. Within ATEP-CMS, the reference fits are computed using the singular value decomposition of the data matrix after translating the centroid to the origin. The properties of the singular value decomposition are well understood, and the magnitude of the error in the direction vector can be bounded using standard techniques [20] as follows.

We consider first the case of line fits. The direction of the line is the right eigenvector corresponding to the largest singular value of the data matrix. The singular value decomposition is computed using C-language double (64 bit IEEE real format) arithmetic, for which the unit roundoff is *ε*_64_ ≈ 2.22×10^−16^. Call the data matrix *D* and denote the exact solution by Q^*^. It can be shown that the computed solution, ***Q***, is (within *ε*_64_) the exact solution to a matrix *D* + *E* where ∥*E*∥_2_ ⩽ *ε*_64_ ∥*D*∥_2_. (Here, ∥·∥_2_ denotes the vector or matrix 2-norm.) From this, it can be shown that [20], as long as the largest singular value is well separated^9^ from the next largest singular value in comparison to ∥*E*∥_2_, the error in the computed solution is bounded by

$${\left\|Q-Q*\right\|}_{2}\leq 4\varepsilon_{64}\frac{\lambda_{1}^{2}}{\left|\lambda_{1}^{2}-\lambda_{2}^{2}\right|}\;(\text{for lines}),$$

where *λ*_1_ is the largest singular value and *λ*_2_ is the next-largest singular value. (This bound is not the tightest possible. For instance, as *λ*_2_ → *λ*_1_, the bound can far exceed the theoretical worst-case difference between ***Q*** and Q* of 2. In such situations, however, the singular values are not well separated. Within ATEP-CMS, the error bound is never allowed to exceed 2.)

Similar results can be obtained for plane fits. In this case, however, the normal to the plane is the right eigen-vector corresponding to the *smallest* singular value, *λ*_3_. The corresponding error bound is

$${\left\|Q-Q*\right\|}_{2}\leq 4\varepsilon_{64}\frac{\lambda_{3}^{2}}{\left|\lambda_{2}^{2}-\lambda_{3}^{2}\right|}\;(\text{for planes}).$$

We have here developed overall bounds for the errors in the eigenvectors. To convert these to standard uncertainties, we assume the errors are uniformly distributed within the bounds. The resulting standard deviation, denoted *u*_D_, is given by

$$u_{D}=\{\begin{array}{ll} \frac{4\varepsilon_{64}\lambda_{1}^{2}}{\sqrt{3}\left|\lambda_{1}^{2}-\lambda_{2}^{2}\right|} & \left(\text{lines}\right)\\ \frac{4\varepsilon_{64}\lambda_{3}^{2}}{\sqrt{3}\left|\lambda_{2}^{2}-\lambda_{3}^{2}\right|} & (\text{planes}). \end{array}$$

These results are quite conservative. In general any one eigenvector will be more sensitive in one direction than another to perturbations in the data. This behavior could, in principle, be used to develop tighter bounds on the numerical errors propagated through the difference computations. Such an analysis will be the subject of future work.

#### 5.1.3 Nonlinear fits

For geometries other than line and plane (circle, sphere, cylinder, cone, and torus), orthogonal distance regression is a nonlinear least-squares optimization problem. (Circle fitting uses plane fitting to reduce the problem to two dimensions.) The algorithm currently used in ATEP-CMS to compute the reference fits is a modified Levenberg-Marquardt algorithm proposed by Nash [21].

The ATEP-CMS algorithm starts with an initial guess and iteratively solves the normal equations for a linear approximation to the nonlinear objective function, where the solution is constrained to lie within a “trust region” of the current best guess. The iterations terminate when the computed solution to the normal equations changes the solution by less than a convergence factor set by the tester. (Typically, the convergence factor used in ATEP-CMS is 10^−15^.)

The uncertainty of the reference fit is a combination of two factors. First, the termination logic introduces an uncertainty that is bounded by the convergence factor setting. Second, the solution to the normal equations at the terminating iteration is subject to numerical round-off effects.

The convergence factor introduces an uncertainty in each element of the computed solution. If the calculated solution is a *k*-dimensional vector ***F***, if F^*^ is the exact solution, and if *C* is the convergence factor, then the resulting error due to *C* alone is bounded by

$${\left\|F-F*\right\|}_{2}\leq C\sqrt{k}.$$

We next deal with the numerical inaccuracies. The fits are calculated using the Choleski decomposition of the normal equations in C-language double arithmetic, so the error in the solution due to numerical roundoff effects alone can be bounded by

$${\left\|F-F*\right\|}_{2}\leq \varepsilon_{64}\kappa (A){\left\|F\right\|}_{2}$$

where *κ* (*A*) is the condition number of the normal equation matrix *A* used for the last iteration of the Levenberg-Marquardt routine, and *ε*_64_ ≈ 2.22×10^−16^ as before [20].

To obtain an overall uncertainty, we must convert these bounds into standard deviations. We assume that the numerical errors and the convergence factor errors are independent and uniformly distributed between the bounds. Denote by *u*_F_ the standard uncertainty for nonlinear fits. Then

$$u_{F}=\sqrt{\frac{C^{2}k}{3}+\frac{\varepsilon_{64}^{2}\kappa^{2}(A){\left\|F\right\|}_{2}^{2}}{3}}.$$

As with linear fits, the bounds developed here bracket the overall behavior of the fit parameter vector. But as with the singular value decomposition, the solution to the nonlinear problem will in general exhibit greater sensitivity and correlations for some parameters than for others. Thus, it may be possible to obtain tighter uncertainty bounds than those developed here. One problem is that, for geometries other than the sphere, the parameterization used for fitting must somehow represent the orientation of the element. Unfortunately, there is no parameterization of orientations that is free of singularities, and there is always the possibility that the Jacobian of the residuals is rank deficient. This makes traditional approaches using, for instance, the condition number of the asymptotic covariance matrix at the computed solution problematic [22]. As with the singular value decomposition, future work will address tightening the bounds on the fit uncertainties.

### 5.2 Propagation to Difference Parameters

This section discusses how the uncertainties of the computed reference fits propagate to the difference parameters for individual fits. As with Sec. 5.1, the results of this section are specific to the particular algorithms currently being used within ATEP-CMS.

The previous section identified several sources of standard uncertainty for the reference fits. We have designated these as follows:

*u*_0_—the standard deviation of the centroid coordinates
*u*_D_—the standard deviation of the error in direction for line and plane fits
*u*_F_—the standard deviation of the error norm of the representation parameter vector for nonlinear fits.

We will separately propagate the standard uncertainties represented by each of these standard deviations through the comparison algorithms and combine the resulting standard uncertainties.

The calculations are fairly straightforward, but tedious. Therefore, we will show details only for the case of the uncertainties of line fits. For other geometry types, we will just present the final results, since the method is the same.

#### 5.2.1 Line

Line differences are defined by two characteristics: the angle between the test and reference lines, and the distance of an endpoint of the test line segment to the reference line. For the angle, we proceed as follows. As in Sec. 5.1.2, let ***Q*** be the direction of the computed reference line and Q* the direction of the true reference line. Also, let ***Q***_T_ be the direction vector of the test fit, α be the angle between ***Q*** and ***Q***_T_, and α* be the angle between ***Q***_T_ and Q*. Since all the direction vectors are unit vectors, the sine of the angle between any two of the vectors is the magnitude of their vector cross-product. We then have:

$$\begin{array}{c} \sin(\alpha )={\left\|Q_{T}\times Q\right\|}_{2}\\ ={\left\|Q_{T}\times (Q*+(Q-Q*))\right\|}_{2}\\ ={\left\|Q_{T}\times Q*+Q_{T}\times (Q-Q*))\right\|}_{2}\\ \leq {\left\|Q_{T}\times Q*\right\|}_{2}+{\left\|Q_{T}\times (Q-Q*))\right\|}_{2}\\ \leq \sin\;(\alpha *)+{\left\|Q-Q*\right\|}_{2}. \end{array}$$

If we start with sin(α*) = ‖***Q***_T_×Q*‖_2_ and follow a similar sequence, we find that sin(α*) ≤ sin(*α*)+ ‖***Q***−Q*‖_2_. Assuming that *α* and α* are small (i.e., that the test and reference results are close), sin(*α*)≈*α* and sin(α*) ≈ α*. Thus, |*α*−α*| ≤ ‖***Q***−Q*‖_2_. Using the results of Sec. 5.1.2, ‖***Q***−Q*‖_2_ is the random variable corresponding to a standard deviation of *u*_D_. We then have

$$u_{\alpha }=u_{D}.$$

We now consider the distance from any point *b* to the reference line. Call this distance *d*. The reference line is located by the centroid of the data, ***q***_0_, so *d* = ‖(***q***−***q***_0_)×***Q***‖_2_. Two components of the fit uncertainty affect the uncertainty of *d*: the uncertainty of ***Q*** and the uncertainty of *q*_0_. We treat these as independent sources of uncertainty and combine them in quadrature. With regard to ***Q***, we proceed as we did with *a* and find that the uncertainty in *d* due to the uncertainty in ***Q*** is bounded by

$${\left\|q-q_{0}\right\|}_{2}{\left\|Q-Q*\right\|}_{2}.$$

The standard uncertainty in *d* due to the standard uncertainty in ***q***_0_ is *u*_0_. Thus, the standard uncertainty of the separation parameter for lines is

$$u_{d}=\sqrt{{u_{0}}^{2}+{\left\|q-q_{0}\right\|}_{2}^{2}{u_{D}}^{2},}$$

where ***q*** is now the endpoint on the test line segment furthest from the reference line.

#### 5.2.2 Circle

The standard uncertainties in the difference parameters for circles are as follows:

- distance between centers: $u_{c}=\sqrt{{u_{0}}^{2}+{u_{F}}^{2}}$
- angle between planes (due to the plane fits): *u_α_* = *u*_D_
- difference in radii: *u*_r_ = *u*_F_.

#### 5.2.3 Plane

The standard uncertainties in the difference parameters for planes are as follows:

- angle between planes: *u_α_* = *u*_D_
- plane separation: $u_{d}=\sqrt{{u_{0}}^{2}+{\left\|q-q_{r}\right\|}_{2}^{2}{u_{F}}^{2}}$, where ***q*** is the point on the test plane furthest from the reference plane and ***q***_0_ is the centroid of the data.

#### 5.2.4 Sphere

The standard uncertainties in the difference parameters for spheres are as follows:

- distance between centers: $u_{c}=\sqrt{{u_{0}}^{2}+{u_{F}}^{2}}$
- difference in radii: *u*_r_ = *u*_F_.

#### 5.2.5 Cylinder

The standard uncertainties in the difference parameters for cylinders are as follows:

- angle between axes: *u_α_* = *u*F
- axis separation: $u_{d}=\sqrt{{u_{0}}^{2}+{\left\|q-q_{r}\right\|}_{2}^{2}{u_{F}}^{2}}$ where ***q*** is the endpoint of the test fit axis furthest from the reference fit axis and ***q***_r_ is the point used to locate the reference fit axis
- difference in radii: *u*_r_ =*u*_F_

#### 5.2.6 Cone

The standard uncertainties in the difference parameters for cones are as follows:

- angle between axes: *u_α_* = *u*F
- axis separation: $u_{d}=\sqrt{{u_{0}}^{2}+{\left\|q-q\right\|}_{2}^{2}{u_{F}}^{2}}$, where ***q*** is the endpoint of the test fit axis furthest from the reference fit axis and ***q***_r_ is the point used to locate the reference fit axis
- difference in axial location: $u_{1}=\sqrt{{u_{0}}^{2}+{u_{F}}^{2}}$
- difference in half-angle: *u*_h_ = *u*_F_

#### 5.2.7 Torus

The standard uncertainties in the difference parameters for tori are as follows:

- angle between planes: *u_α_* = *u*_F_
- center separation: $u_{c}=\sqrt{{u_{0}}^{2}+{u_{F}}^{2}}$
- difference in major radii: *u*_M_ = *u*_F_
- difference in minor radii: *u_m_* = *u*_F_

### 5.3 Uncertainty of the Estimated Performance Parameters

The results of Sec. 5.2 provide us with a means of estimating the uncertainties, expressed as standard deviations, of individual difference parameters *p_i_* for individual data sets. Using this, we can now estimate the uncertainty of the software performance measures for each geometry type.

Recall (from Sec. 4.2) that for a particular performance characteristic, we are estimating the performance measure by the observed root-mean-square value of the difference parameters:

$${\hat{\gamma }}_{P}=\sqrt{\frac{1}{n}\sum {p_{i}}^{2}}.$$

Furthermore, we have an estimate, denoted by *u_i_*, for the standard deviation of each *p_i_* attributable to the uncertainty of the reference fit.

Our objective is to estimate the variance of ${\hat{\gamma }}_{P}$. To that end, we will use the following identity:

$$V({\hat{\gamma }}_{P})=E_{A}[V({\hat{\gamma }}_{P}|A)]+V_{A}[E{\hat{\gamma }}_{P}|A)],$$

where *A* is a random event, $E({\hat{\gamma }}_{P}|A)$ and $V({\hat{\gamma }}_{P}|A)$*A*) are, respectively, the expected value and variance of ${\hat{\gamma }}_{P}$ conditional on event *A*, and *E_A_*[ ] and *V_A_*[ ] are, respectively, the expected value and variance over all events *A*. This identity, which follows from the theorem of total probability, helps us estimate the variance of ${\hat{\gamma }}_{P}$ by using the observed sample of performance for the data sets we used.

Let *A* be the event that the sample of observations is the one we indeed observed. (By conditioning on *A* we are treating the sample as fixed and not random.) We can interpret the two terms of the variance as follows. The first term, $E_{A}[V({\hat{\gamma }}_{P}|A)]$ represents the component of standard uncertainty due to the uncertainty of the reference fits. We will denote this quantity by *u*_R_^2^, meaning “square of the standard uncertainty due to the reference.” The second term, $V_{A}[E({\hat{\gamma }}_{P}|A)]$, represents the component of standard uncertainty due to the variation in the sampling. We will denote this by *u*_S_^2^, meaning “square of the standard uncertainty due to sampling.” We now develop estimates for the two components of uncertainty, starting with *u*_R_.

#### 5.3.1 Uncertainty Due to the Reference

To estimate *u*_R_, we must first estimate $V({\hat{\gamma }}_{P}|A)$. Considering ${\hat{\gamma }}_{P}$ as a function of the *p_i_*, we have, using the law of propagation of uncertainty

$$V({\hat{\gamma }}_{P}|A)\approx \sum {\left[\frac{\partial {\hat{\gamma }}_{P}}{\partial p_{i}}\right]}^{2}{u_{i}}^{2}=\frac{\Sigma {p_{i}}^{2}{u_{i}}^{2}}{n\Sigma {p_{i}}^{2}}.$$

(If all the *p_i_* are zero (as might happen, for example, if the NIST algorithms are tested against themselves), then this estimate is indeterminate. However, whenever *p_i_* is small in relation to *u_i_*, we can improve the estimate by evaluating the partial derivatives in the propagation formula at the expected value of |*p_i_*| instead of at the expected value of *p_i_*. To do this, we assume that *p_i_* is distributed uniformly about the observed value within $\pm \sqrt{3}u_{i}$. Then, whenever $|p_{i}|<\sqrt{3}u_{i}$ the mean should be modified to include the “folding back” at zero of the distribution of |*p_i_*|. The result is

$$V({\hat{\gamma }}_{P}|A)\approx \frac{\Sigma {{\tilde{p}}_{i}}^{2}{u_{i}}^{2}}{n\Sigma {{\tilde{p}}_{i}}^{2}}$$

where

$${\tilde{p}}_{i}=\{\begin{array}{ll} p_{i} & \text{if}\;{p_{i}}^{2}\geq 3{u_{i}}^{2}\\ \frac{{p_{i}}^{2}+3{u_{i}}^{2}}{2\sqrt{3}u_{i}} & \text{if}\;{p_{i}}^{2}<3{u_{i}}^{2} \end{array}$$

This approximation to $V({\hat{\gamma }}_{P}|A)$ is used in ATEP-CMS as an estimate of $E_{A}[V({\hat{\gamma }}_{P}|A)]$

$${{\hat{u}}_{r}}^{2}=\frac{\Sigma {{\tilde{p}}_{i}}^{2}{u_{i}}^{2}}{n\Sigma {{\tilde{p}}_{i}}^{2}}$$

(If all the ${\tilde{p}}_{i}$ are zero—that is, *u_i_* = 0 whenever *p_i_* = 0— then we set ${\hat{u}}_{R}=0$.)

The component of standard uncertainty due to the reference is estimated from numerical analysis considerations and, in the language of the guidelines on uncertainty [11, 10], is a Type B standard uncertainty.

#### 5.3.2 Uncertainty Due to Sampling Variation

We now turn to *u*_S_^2^, the component of standard uncertainty due to sampling. To estimate *u*_S_^2^, we need to estimate $E({\hat{\gamma }}_{P}|A)$. We do this by simply using a zero-order expansion of ${\hat{\gamma }}_{P}$ about the *p_i_*, giving $E({\hat{\gamma }}_{P}|A)\approx {\hat{\gamma }}_{P}$, and we are left with estimating $V_{A}({\hat{\gamma }}_{P})$. If ${\hat{\gamma }}_{P}=0$, we estimate $V_{A}({\hat{\gamma }}_{P})=0$. Otherwise we proceed as follows. By the law of propagation of uncertainty,

$$V_{A}(\hat{\gamma })\approx \frac{1}{4{{\hat{\gamma }}_{P}}^{2}}V_{A}({{\hat{\gamma }}_{P}}^{2})=\frac{1}{4n^{2}{{\hat{\gamma }}_{P}}^{2}}\sum V_{A}({p_{i}}^{2}).$$

For convenience, we denote by *σ*_A_^2^ the mean variance of the *p_i_*^2^ over all samples

$${\sigma_{A}}^{2}=\frac{1}{n}\sum V_{A}({p_{i}}^{2}).$$

Since ${\hat{\gamma }}_{P}$ is estimated from the sample, an unbiased estimate for *σ*_A_^2^ is:

$${{\hat{\sigma }}_{A}}^{2}=\frac{1}{n-1}\sum {\left({p_{i}}^{2}-{{\hat{\gamma }}_{P}}^{2}\right)}^{2}.$$

We then have

$${{\hat{u}}_{S}}^{2}=\frac{\Sigma {\left({p_{i}}^{2}-{{\hat{\gamma }}_{P}}^{2}\right)}^{2}}{4n(n-1){{\hat{\gamma }}_{P}}^{2}}=\frac{\Sigma {\left({p_{i}}^{2}-{{\hat{\gamma }}_{P}}^{2}\right)}^{2}}{4(n-1)\Sigma {p_{i}}^{2}},$$

with ${\hat{u}}_{S}=0$ if all of the *p_i_* = 0.

Since the component of uncertainty due to sampling is estimated from a statistical analysis of data, it is a Type A standard uncertainty, with *n*−1 degrees of freedom.

#### 5.3.3 Combined Standard Uncertainty

Putting the two terms of the variance identity together, the combined standard uncertainty of the estimated performance parameter ${\hat{\gamma }}_{P}$ is the positive square root of the estimated variance of ${\hat{\gamma }}_{P}$

$$V({\hat{\gamma }}_{P})={u_{R}}^{2}+{u_{S}}^{2}\approx \frac{\Sigma {{\tilde{p}}_{i}}^{2}{u_{i}}^{2}}{n\Sigma {{\tilde{p}}_{i}}^{2}}+\frac{\Sigma {\left({p_{i}}^{2}-{{\hat{\gamma }}_{P}}^{2}\right)}^{2}}{4(n-1)\Sigma {p_{i}}^{2}}.$$

Following NIST convention, the expanded uncertainty reported by ATEP-CMS is twice the combined standard uncertainty (i.e., a coverage factor of *k* = 2).

## 6. Summary

This paper has described how NIST evaluates the performance of geometric fitting software used for inspection. The NIST service, ATEP-CMS, is the only known test that provides quantitative measures of performance, complete with statements of uncertainty in accordance with international standards.

ATEP-CMS is something new in the way of calibration services. It is the only test of software offered by NIST in the field of dimensional metrology. It has the status of a Special Test, rather than a Calibration Service, because it is an experiment for us. The statements of uncertainty, in particular, are unsupported by any historical data. We believe, however, that they are fundamentally sound. We have tested some of our capabilities by running our software on both a personal computer and on NIST’s supercomputer. These limited results support the validity of our approach.

This paper has focused on the performance measures used in ATEP-CMS; we have not discussed testing procedures. However, one key aspect of those procedures bears discussion: the selection of data sets to be used for the test. We use the collection of data sets as if the average performance of the software over those problems represents the average performance of the software when it is used in production. To help ensure this, we use a stratified sampling approach in designing data sets for a test. Through judgment, we define ranges of parameters for inspection problems, including ideal geometry, form errors, surface sampling plans, and point measurement errors. Within each range, we select a representative sampling of data sets. We believe that this mixed approach improves the quality of the test.

We expect to refine our procedures as we gain experience in testing. We also plan to extend the scope of our testing services beyond orthogonal distance regression to include other fitting methods and other common CMS software functions [23].

The methods described in this paper demonstrate that classical concepts of metrology can be used to assess the performance of software, when that software forms part of a measurement system. Moreover, it seems that performance testing like that offered by ATEP-CMS is a requirement if CMS measurements are to be traceable to accepted standards of length.

## Footnotes

1 ATEP-CMS—part of a larger field of endeavor called *computational metrology* [2]—addresses the *fitting software* as a source of uncertainty due to possible computational errors. It does not address the propagation of point coordinate uncertainty through the geometric fitting computations, how well the points represent the surface, or whether the intended software function is the most appropriate for the task.

2 Orthogonal distance regression is commonly called “least squares” by manufacturing practitioners. Statisticians consider these two terms to be quite different. Lease squares measures deviations from the surface along a particular direction in a coordinate system. Orthogonal distance regression (which is also called “errors in variables” regression or, for linear fits, “total least squares”) measures deviations perpendicular to the surface. Unfortunately, some of the literature (e.g., Ref. [8]) further confuses the terminology by using the term “orthogonal distance regression” for one specific algorithm that solves the orthogonal distance regression problem.

3 A small disclaimer is in order here. The use of the term “reference fit” is not meant to imply that the ATEP-CMS fitting algorithms are “standard algorithms” for geometric fitting problems. For instance, the algorithm used in ATEP-CMS for nonlinear orthogonal distance regression (see Sec. 5.1.3) was chosen for its efficient use of memory, but its use of normal equations in the iteration are a known weakness. Algorithms with superior numerical properties are widely available (see, e.g., Refs. [12, 8]) and may be used by ATEP-CMS in the future.

4 For instance, it can be used to detect if something other than the two-step fitting process was used by the software under test.

5 See, e.g., Ref. [15, Chap. 2].

6 This assumption will be tested using data collected during future operation of ATEP-CMS.

7 Throughout this paper, a carat appearing over a quantity denotes an estimated value.

8 Other errors, such as floating-point roundoff during evaluation of the difference parameters, are considered small enough to be ignored.

9 The notion of “well separated” can be made precise. Essentially, it means that the data has one dominant direction of scatter (so a line fit makes sense).
